# Showing their true colors: a practical approach to volume rendering from serial sections

**DOI:** 10.1186/1471-213X-10-41

**Published:** 2010-04-21

**Authors:** Stephan Handschuh, Thomas Schwaha, Brian D Metscher

**Affiliations:** 1 Department of Theoretical Biology (Gerd Müller, Head), University of Vienna, Althanstrasse 14, 1090 Vienna, Austria

## Abstract

**Background:**

In comparison to more modern imaging methods, conventional light microscopy still offers a range of substantial advantages with regard to contrast options, accessible specimen size, and resolution. Currently, tomographic image data in particular is most commonly visualized in three dimensions using volume rendering. To date, this method has only very rarely been applied to image stacks taken from serial sections, whereas surface rendering is still the most prevalent method for presenting such data sets three-dimensionally. The aim of this study was to develop standard protocols for volume rendering of image stacks of serial sections, while retaining the benefits of light microscopy such as resolution and color information.

**Results:**

Here we provide a set of protocols for acquiring high-resolution 3D images of diverse microscopic samples through volume rendering based on serial light microscopical sections using the 3D reconstruction software Amira (Visage Imaging Inc.). We overcome several technical obstacles and show that these renderings are comparable in quality and resolution to 3D visualizations using other methods. This practical approach for visualizing 3D micro-morphology in full color takes advantage of both the sub-micron resolution of light microscopy and the specificity of histological stains, by combining conventional histological sectioning techniques, digital image acquisition, three-dimensional image filtering, and 3D image manipulation and visualization technologies.

**Conclusions:**

We show that this method can yield "true"-colored high-resolution 3D views of tissues as well as cellular and sub-cellular structures and thus represents a powerful tool for morphological, developmental, and comparative investigations. We conclude that the presented approach fills an important gap in the field of micro-anatomical 3D imaging and visualization methods by combining histological resolution and differentiation of details with 3D rendering of whole tissue samples. We demonstrate the method on selected invertebrate and vertebrate specimens, and propose that reinvestigation of historical serial section material may be regarded as a special benefit.

## Background

Understanding developmental processes in this age of sophisticated genetic and functional analyses depends just as much on accurate knowledge of microscopic anatomy as it did in the time of classical embryology. Today, a broad assemblage of analysis and visualization techniques is used to generate, revise, and re-evaluate morphological data. Modern 3D imaging and visualization methods are yielding new insights by displaying morphological structures as well as gene expression patterns to micron or sub-micron resolutions (reviewed in [1]). Recently, different tomographic technologies have been applied to image soft-body parts and gene expression in small biological samples. X-ray microtomography (microCT) yields resolutions usually down to a few microns, ideal for imaging embryos [2] and other small specimens [3], but x-ray absorbances of soft tissues are extremely low. Heavy-metal containing contrast agents provide good contrast in organic tissues [2,4-6], but so far no methods are available for specific staining of tissues or gene products. Microscopic magnetic resonance imaging (microMRI) enables high-contrast imaging of untreated biological specimens [7-9], and, with special genetic constructs, can also be used to image changing gene expression patterns in living specimens [10]. However, the main drawback of microMRI is a physical resolution limit of approximately 10 μm, significantly higher in practice [11]. Optical projection tomography (OPT) can yield higher resolutions and since 3D images are obtained through absorption or emission of visible light, this method can use the variety of specific optical colored or fluorescent stains to visualize tissues and gene expression patterns [11,12]. Confocal laser scanning microscopy (CLSM) differs from these projection-tomographic techniques, since 3D images are gained through generating optical sections through an object, described also as section tomography. CLSM currently supplies a substantial portion of novel morphological findings at histological scales. Whole-mount fluorescence preparations using specific markers such as anti-serotonin (nervous tissue) or F-actin (musculature) enable high-resolution 3D visualizations of specific tissues, but are usually limited to an object thicknesses of approximately 100 μm (for a review see [13]). However, special technical setups can be used to image larger tissue samples by CLSM [14]. Reconstruction of serial physical sections represents the oldest method of presenting micro-anatomical data three-dimensionally, and manual reconstruction methods date back to the late nineteenth century [15-18]. In comparison to modern analysis methods, conventional light microscopical sections still offer a range of benefits with regard to e.g. contrast options or accessible specimen size. Only few of the modern 3D techniques, such as synchrotron-based microCT or CLSM, can compete with light microscopical sections in terms of sub-micron resolution. Although the technical procedure of physical sectioning continues to be comparatively laborious, recent progress in light microscopical sectioning techniques [19,20], as well as automatic block-face image capturing methods like episcopic fluorescence image capturing (EFIC) or surface imaging microscopy (SIM), have dramatically increased the efficiency and accuracy of serial reconstructions [21,22].

For the past decade, volume rendering (VR) has represented one of the most widely applied methods to visualize tomographic data three-dimensionally. In VR an image projection is constructed by simulating the absorption and emission of light in an image stack along each ray path to the eye (introduced by [23]). By this process brightness and color of each voxel (volumetric pixel) is calculated from its gray or color value and from its transparency (determined by an assigned transparency function). To date, VR has only rarely been applied to image stacks taken from light microscopical sections [24,25]; surface rendering based on image segmentation is still the most prevalent method for presenting histological data three-dimensionally (for examples see [26-30]).

In the approach presented here, 3D visualizations are obtained through VR based on different types of recent and historical serial physical sections. The aim of this study was to develop standard protocols that yield 3D images of a quality and resolution comparable to recent 3D imaging methods and that also take advantage of the variety and specificity of available histological stains.

## Methods

### Animals

To demonstrate the broad range of application of the presented method we have chosen to use section series of several different invertebrate and vertebrate taxa. The freshwater bryozoan *Cristatella mucedo *Cuvier 1798 (collected from the Laxenburg Pond, Lower Austria) and the phoronid *Phoronis australis *Haswell 1883 (unknown sampling site) were selected as invertebrate representatives for a comparison of different embedding media. An embryo of the Madagascan Nile crocodile, *Crocodylus niloticus madagascariensis *Grandidier 1872, collected in Madagascar during a field trip in the late nineteenth century, was chosen as example for vertebrate tissue.

### Histological treatment

*Cristatella mucedo *colonies were anesthetized with chloral hydrate, fixed in Bouin's solution, dehydrated with acidified dimethoxypropane and embedded in low-viscosity resin (Agar Scientific Ltd., Stansted, UK) using acetone as intermedium. Ribboned semithin serial sections of 1 μm section thickness were made with a Histo-Jumbo diamond knife (Diatome AG, Biel, Switzerland) on a Reichert UltraCut-S microtome (for further technical details see also [20]). Sections were stained with toluidine blue [31] at 60°C for 30 seconds.

For the *Phoronis australis *sample, details of fixation are unknown. It was embedded in paraffin wax, sectioned with a section thickness of 7 μm and stained using Heidenhain's azan trichrome stain [31,32].

The specimen of *Crocodylus niloticus madagascariensis *was most likely fixed and preserved in ethanol. For a comparative investigation on Sauropsidian lower jaws [33,34] it was embedded in paraffin wax in the late 1970s, sectioned at a thickness of 14 μm and stained using light-green orange-G stain [35]. Due to its advanced age, this specimen exemplifies the applicability of the presented method on historical serial section material.

### Digital image acquisition and editing

Digital images were manually captured on an Axio Imager A1 microscope (Carl Zeiss MicroImaging GmbH, Göttingen, Germany) using a ProgRes C14 camera (Jenoptik, Jena, Germany) at an image resolution of 4080 × 3072 pixels, and saved in uncompressed TIFF format at 24-bit RGB color depth. Subsequently, prior to 3D processing, the section images were edited using Adobe Photoshop CS3. First, obvious artifact such as dust or staining precipitates were removed from the image background using the tools *brush *and *clone stamp*, mainly to reduce background noise in the final 3D images. Next, they were contrast-enhanced (additionally, the background of all images was set to the white point to obtain a completely homogenous white background throughout the image stack), cropped to a region of interest, and image size was reduced to approximately one megapixel. In resizing section images, we have set the final image resolution so that the ratio of section thickness to pixel edge-length did not exceed 3:1. Higher ratios yield more elongate voxels and obviously lead to anisotropic 3D visualizations. Ideally, voxels are exactly cubic. For further 3D procedures, all image stacks were color inverted and stored both in 8-bit grayscale and 24-bit RGB uncompressed TIFF format.

### Image alignment

Serial section images were loaded into the program Amira 4.1 (Visage Imaging Inc., Andover, MA) using the import functions *Channel 1 *for 8-bit grayscale and *ColorField *for 24-bit RGB image sequences, and automatically aligned using the *least-squares *function of the Amira *AlignSlices *tool. Automatically aligned image stacks were checked by eye and corrected manually if required. After alignment the image stacks were saved in Amira mesh (AM) format.

### Digital 3D image filtering in grayscale image stacks

Paraffin wax sections are prone to small differences in section stretching. To reduce uneven stretching artifacts in the final visualization, two kinds of image filters were applied on aligned image stacks. First, the Amira 3D Gauss filter (6 × 6 × 6 kernel, sigma = 1, 1, 1) was applied for general smoothing. This balances inhomogeneous stretching by averaging gray values, performing a three-dimensional convolution with a Gaussian kernel. Consequently, structures and borders in less-stretched sections are interpolated according to the gray values in neighboring sections. In a second smoothing step we applied the Amira 3D edge-preserving-smoothing filter using the default parameters (contrast = 3.5, sigma = 3, step = 5, stop = 25). This filter again smooths the image stack but also prevents a blurring of edges by varying the kernel depending on image content.

**Figure 1:**
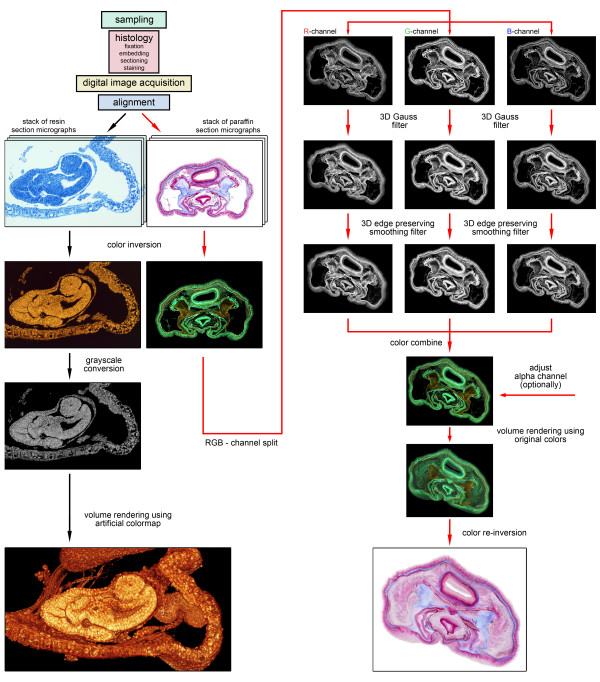
**Flow diagram for volume rendering from serial sections**. Flow diagram showing volume rendering protocols for grayscale (black pathway) and colored (red pathway) image stacks using the 3D software package Amira 4.1. Since the presented grayscale image stack of *Cristatella mucedo *is based on resin sections, image filtering is not explicitly necessary and VR can be performed directly on the aligned image stack. The presented colored image stack of *Phoronis australis *is based on paraffin wax sections, thus the pre-rendering procedure is more complex, including the application of two different 3D image filters on the single RGB channels of the stack.

Image stacks based on resin sections were rendered either directly without application of image filters (as seen in Figure 1, black pathway), or using a modest three-dimensional smoothing with the Amira 3D Gauss filter (3 × 3 × 3 kernel, sigma = 1, 1, 1).

### Digital 3D image filtering in RGBA color image stacks

Since the described image filters cannot be applied directly to color image stacks, each colored image stack was split in its three RGB channels prior to filtering. To split RGB channels, the RGBA AM file was saved as 2D TIFF image sequence. These images were then re-imported into Amira, but this time choosing the import function *AllChannels*. At this point, the aforementioned 3D image filters were applied using same parameters for each RGB channel separately. After filtering, the RGB channels were again combined into a single RGBA stack using the tool *ColorCombine *(as shown for paraffin wax section images in Figure 1, red pathway).

### Adjusting the alpha-channel in RGBA color image stacks

In Amira, RGBA image stacks (AM files) are composed of three color channels (R, G, B) and a fourth transparency channel (A, alpha). Voxels that possess a high (bright) alpha value appear opaque in the subsequent VR, whereas voxels with a low (dark) alpha value are displayed as transparent. It was necessary to adjust this alpha channel so that only object voxels are visible in the final rendering and background voxels are absolutely transparent. This was achieved by replacing the default alpha channel (which is completely white after importing a colored image sequence) by a new alpha channel computed by grayscale conversion using the standard weighting equation "gray value = 0.3 R + 0.59 G + 0.11 B" (also commonly referred as the NTSC formula for luminance) from the three 3 RGB channels. This channel was computed using the Amira *CastField *tool (Output Datatype: unsigned char (8 bit); Color Channel: Channel 4 (grey)). Next this gray channel was incorporated as the alpha channel in the RGBA stack using the *ChannelWorks *tool (Input 1: RGBA stack; Input 2: gray (alpha) stack; Input 1 channel 1 (red) = new channel 1, Input 1 channel 2 (green) = new channel 2, Input 1 channel 3 (blue) = new channel 3, Input 2 channel 1 = new channel 4 (alpha)). Alternatively one can also import an already-grayscale-converted version of the stack (Photoshop uses the same weighting equation for grayscale conversion), or import the colored image sequence a second time, this time choosing the import function *Luminance*. Incorporating the corresponding grayscale stack as alpha channel results in a linear transparency function (color values to alpha values) and in almost total transparency in the object background, since in the three RGB channels and consequently in the converted grayscale channel the background is black (based on the performed color inversion). After replacement of the alpha channel the image stack was again saved as RGBA AM file. However, if a color stack is split into the three RGB channels, filtered, and finally re-combined using the *ColorCombine *tool, the alpha channel is automatically set to the corresponding gray channel during this step. Consequently, no manual adjustment is necessary to obtain a linear transparency function based on the corresponding gray channel.

### Volume rendering

**Figure 2:**
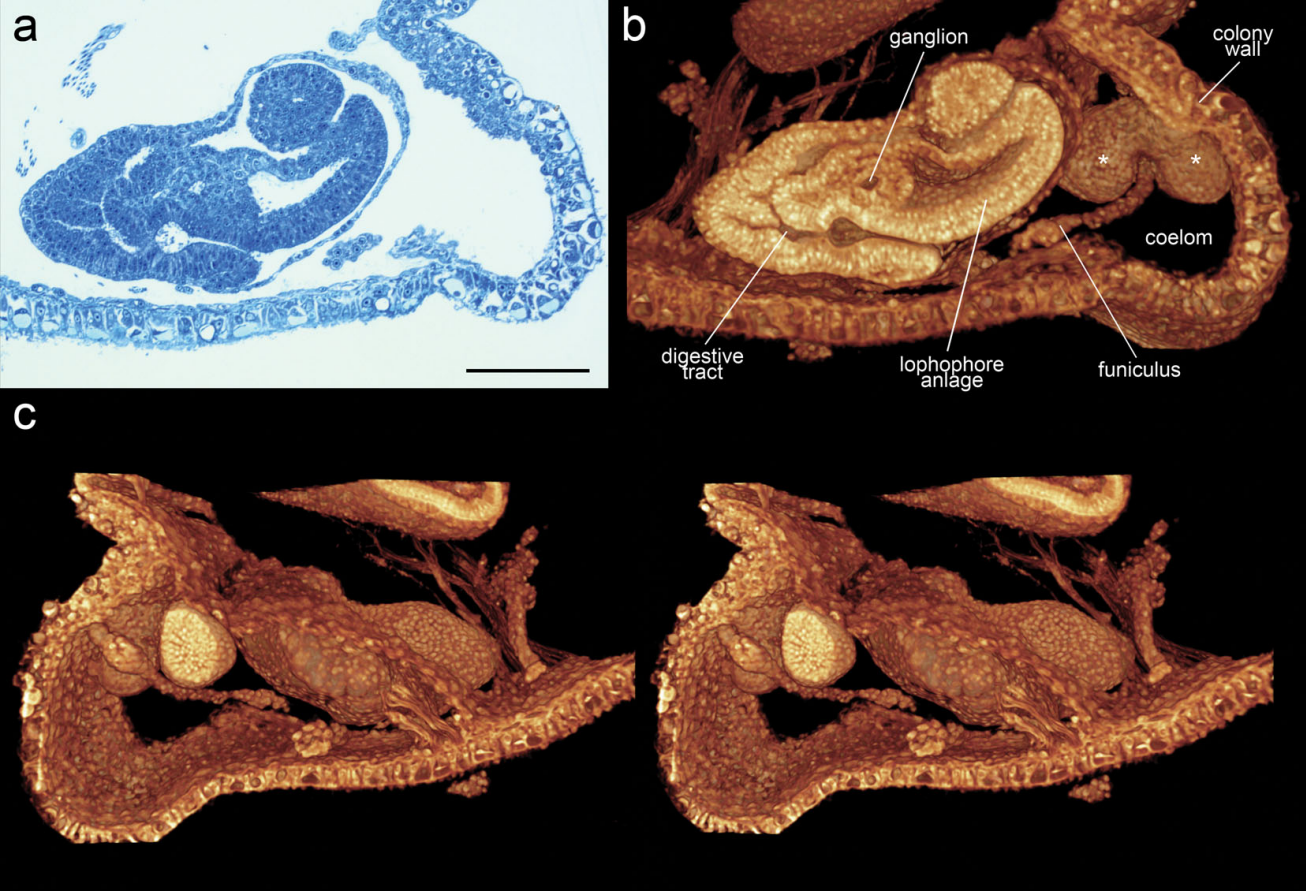
**Volume rendering of a grayscale image stack based on resin sections of the freshwater bryozoan *Cristatella mucedo***. (a) Original section image, toluidine blue staining (200× magnification). (b) Volume rendering based on 254 grayscale section images using the Amira 4.1 *Voltex *tool (artificial *glow *colormap, linear transparency function). *white asterisks = young developing buds*. (c) Stereo image from opposite view of 2 b. Scale bar = 100 μm.

**Figure 3:**
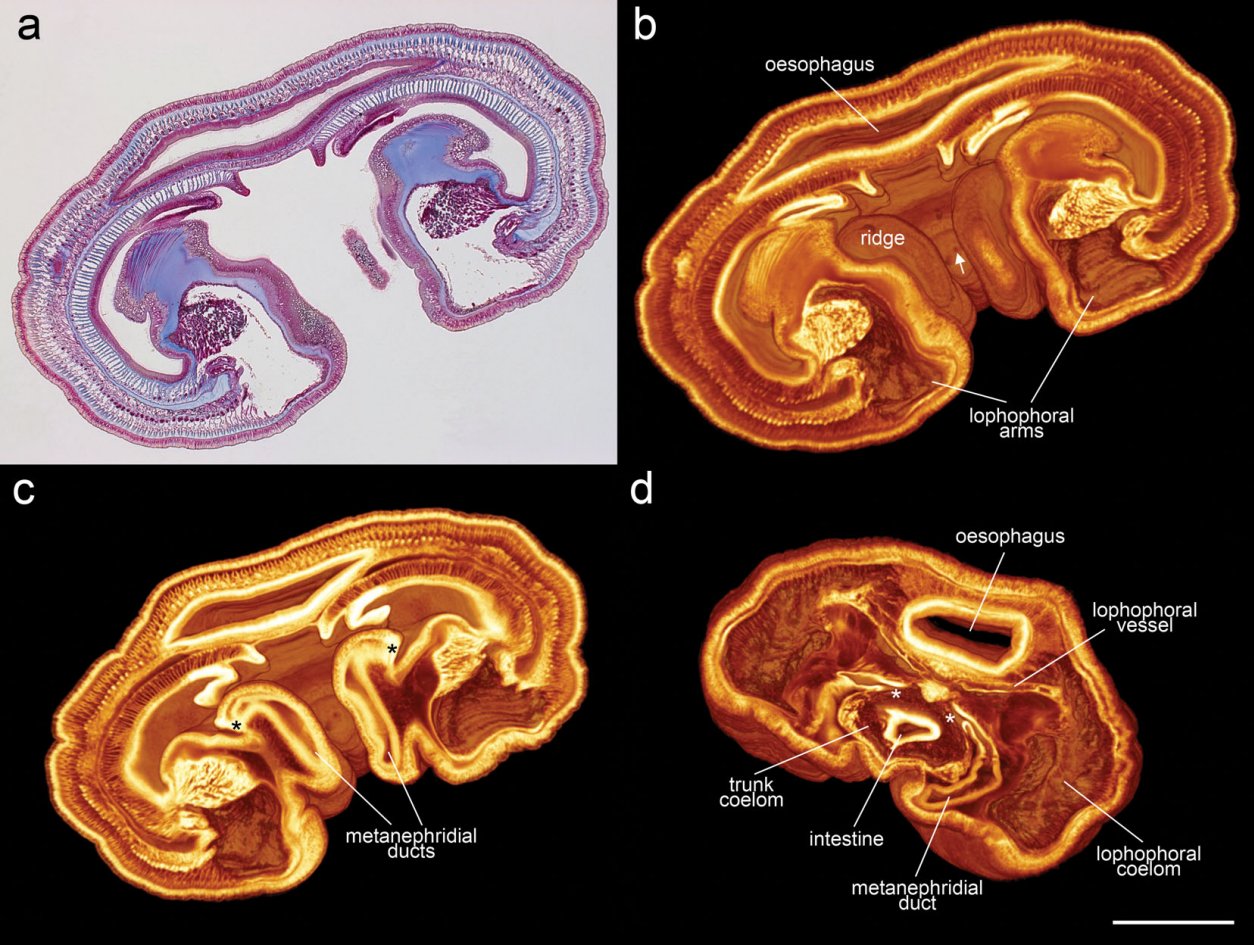
**Volume rendering of a grayscale image stack based on paraffin wax sections of the phoronid *Phoronis australis***. (a) Original section image, Heidenhain's Azan trichrome stain (40× magnification). (b) Volume rendering based on 115 grayscale section images using the X-Radia TXM-viewer (artificial *glow *colormap, linear transparency function). *white arrow = anus*. (c) The same volume rendering as in 3 b but further trimmed. *black astersisks = pores of metanephridial ducts*. (d) View from the adult posterior end. *white asterisks = funnel openings of metanephridia*. Scale bar = 500 μm.

**Figure 4:**
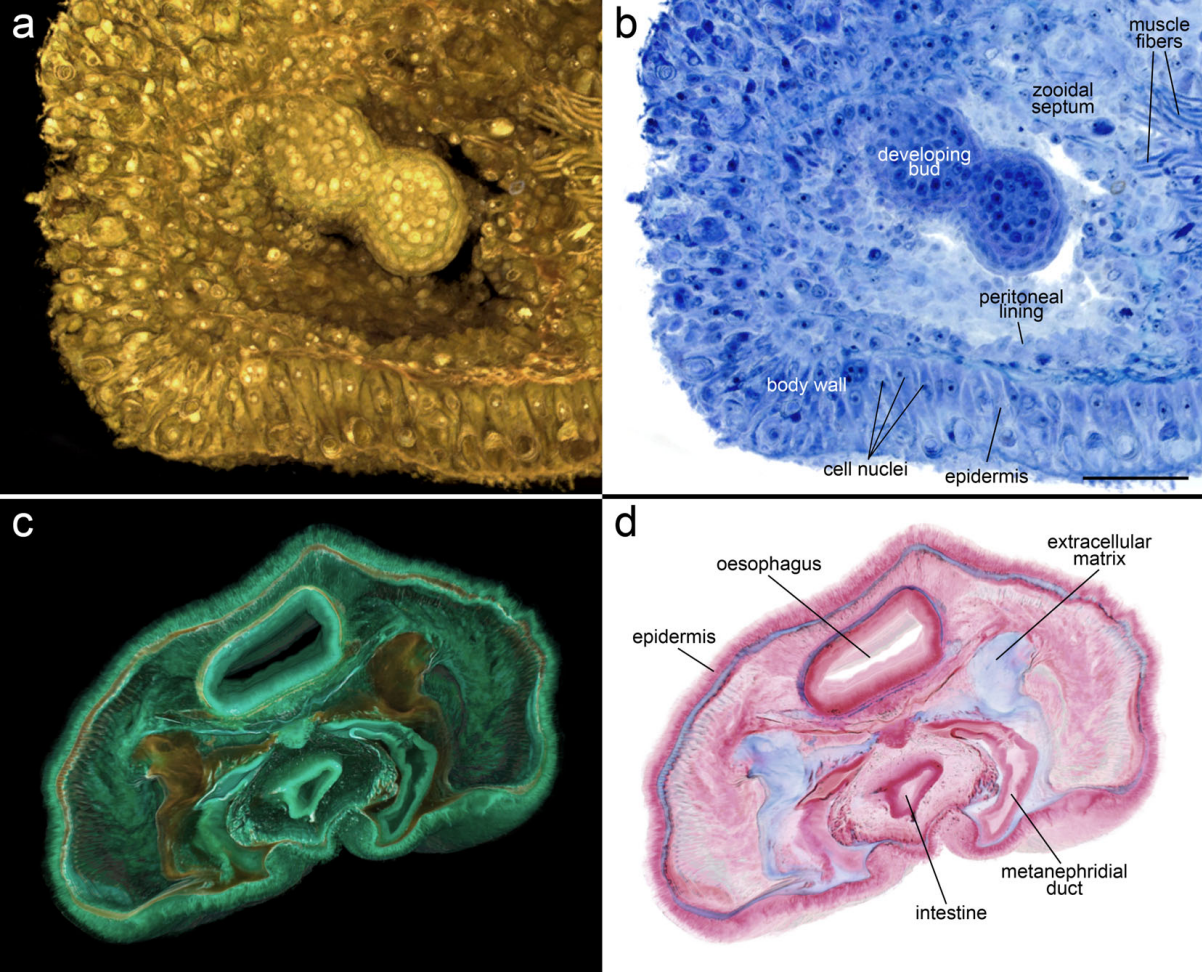
**Examples of volume rendering based on the true colors of the histological stain**. (a) Amira volume rendering of a RGBA stack based on 55 inverted section images taken from toluidine blue-stained resin sections of an early *Cristatella mucedo *bud. Because of color-inversion the background is black, and by using the inverted grayscale channel as alpha channel we achieved total transparency in these background voxels. (b) The re-inverted snapshot of the same volume. Through re-inversion the volume can be visualized using the original colors of the toluidine blue stain. (c) Amira volume rendering of a RGBA stack based on 115 inverted section images taken from Azan-stained paraffin wax sections of *Phoronis australis*. (d) The re-inverted snapshot of the same volume visualized using the original colors of Heidenhain's Azan trichrome stain. Scale bar for a and b = 50 μm. Scale bar for c and d = 500 μm.

For VR of grayscale image stacks we mainly used the Amira 4.1 *Voltex *tool (Figure 2). This volume viewer tool was linked to a *ROI *(region of interest) tool for cropping the volume visualization, as well as to a *Colormap *tool for manual adjustment on colors and transparency function. Note that the *ROI *tool has to be first created from the image stack (*Display*: *ROI*), before linking the *Voltex *to it. For most specimens slightly modified versions of the default *glow *colormap (*volrenGlow*) with an almost linear transparency function were applied for rendering. In addition to the Amira *Voltex *tool we used a volume viewer called TXM-Viewer (supplied with the x-ray microCT system from XRadia Inc., Concord, CA), which also gives various options for adjusting volume visualizations (Figure 3). For VR of colored RGBA image stacks we exclusively used the Amira 4.1 *Voltex *tool (Figure 4) combined with the *ROI *tool.

## Results

### 1 Volume rendering based on grayscale image stacks

#### 1.a: Semithin resin sections of *Cristatella mucedo*

*Cristatella mucedo *are elongated, colonial, freshwater bryozoans of gelatinous consistency. All gelatinous Phylactolaemates possess tightly packed individual zooids. In *C. mucedo *new zooids develop by asexual budding along the colony margin, as seen in an original section image (Figure 2a). The bottom of the colony wall (gliding sole) continues in the lateral colony wall that represents the budding zone from which young developing buds continuously arise. The illustrated developmental stage (254 sections) already shows most morphological features of adult bryozoans: the lophophore anlage, a U-shaped digestive tract, a ganglion and the funiculus. The thin peritoneal lining covering the developing tissues resolved down to the level of cell nuclei is visible. Short developing retractor muscle fibers attach at various sites of the growing polypide. Behind the oldest bud, muscle fibers can be seen traversing the coelom and attaching to the polypide of a full-grown zooid (Figure 2b). A stereo image shows the same developing bud from the opposite side (Figure 2c) (for further details on freshwater bryozoan development and morphology see [36,37]).

#### 1.b: Paraffin wax sections of *Phoronis australis*

*Phoronis australis *(phylum Phoronida) is a solitary species that is notably larger than the individual zooids of the bryozoan *Cristatella mucedo*. Like bryozoans, the phoronid bauplan is characterized by voluminous coelomic cavities. VR of the selected body region (115 sections), at the border of the trunk and lophophoral coelom (an original section image is shown in Figure 3a), shows several morphological structures such as metanephridia and blood vessels. If viewed from the adult anterior end (Figure 3b), the bases of the two lophophoral arms (containing the lophophoral coelom), the cross-sectioned oesophagus, and the anus can be clearly observed. On both sides of the anus, the nephridial ridges indicate the position of the metanephridial ducts inside the animal. If the same VR is further cropped (Figure 3c), the lumen of the metanephridial ducts and their pores to the exterior medium become visible. Viewing from the opposite (adult posterior) side (Figure 3d) the three-dimensional arrangement of the proximal part of the lophophoral coelom as well as the cross-sectioned oesophagus and the intestine are clearly distinguishable. Also displayed are the funnel openings of metanephridia to the trunk coelom, and the median blood vessel bifurcating into two descending lophophoral blood vessels (for more detailed descriptions of phoronid morphology see also [38-40]).

### 2 Volume rendering based on RGBA color image stacks

#### 2.a: Semithin resin sections of *Cristatella mucedo*

Bryozoan buds arise as proliferations or invaginations of the epidermis and the underlying peritoneal layer. In the present visualization (based on 55 sections) of the illustrated early bud, both cell layers have protruded from the colony margin into the coelomic cavity of the colony. Nuclei and even nucleoli are clearly distinguishable in the outer budding layer as well as in the colony wall. Next to the bud, muscle fibers and incomplete septa separating the individual zooids in the colony traverse the coelom of the compact colony (Figures 4a and 4b).

#### 2.b: Paraffin wax sections of *Phoronis australis*

The same image sequence of *Phoronis australis *used to produce Figure 3 was used for full-colored RGBA volume rendering (115 sections). By preserving the color contrasts of Heidenhain's azan trichrome stain, the true-histological colored VR easily distinguishes different tissues. The epidermis and the linings of the digestive tract, metanephridia, and peritoneum are displayed in pink and purple tones. Basal laminae and extracellular matrices appear in various tones of blue, and muscles, and blood cells appear in strong red (Figures 4c and 4d; these figures show exactly the same view as Figure 3d). Due to color contrast afforded by slight staining of the extracellular matrix, the latter is clearly distinguishable in the true-color rendering (Figure 4d), whereas it can hardly be recognized in a rendering of a grayscale stack (Figure 3d).

### 3 Volume rendering based on historical serial sections, exemplified by *Crocodylus niloticus madagascariensis*

**Figure 5:**
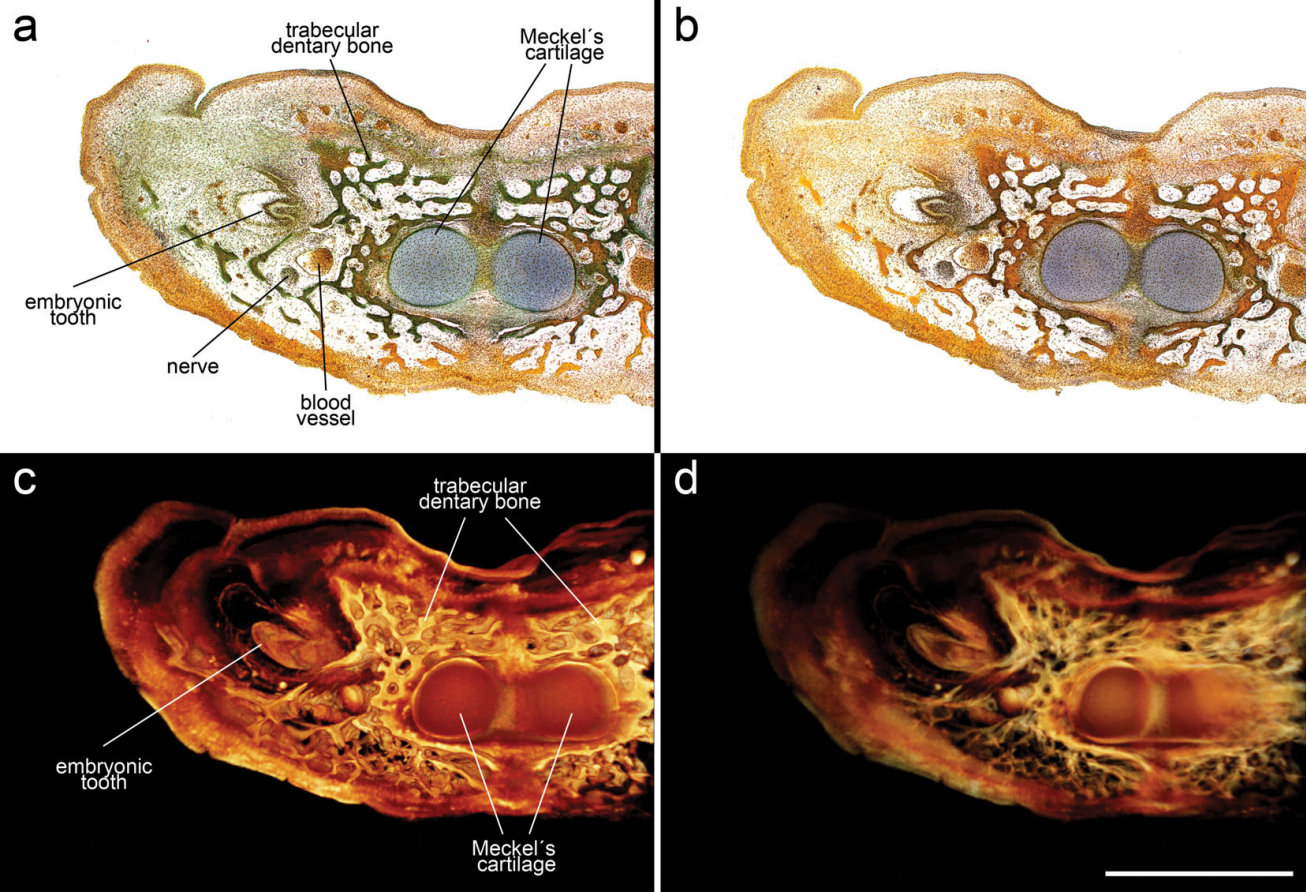
**Volume rendering of historical serial sections showing an embryonic lower jaw of *Crocodylus niloticus madagascariensis***. (a) Original section image with small bleaching artifacts (40× magnification), staining after Halmi [35]. (b) Original section image with notable bleaching artifacts in the light green stain, whereas the orange-G stain still persists all through the object. (c) Volume rendering based on 42 grayscale section images. (d) Volume rendering based as in (c) but with a higher transparency. Scale bar = 500 μm.

The developmental stage treated here lies in between stages 62 and 63 from Voeltzkow [41]. The selected region for rendering (42 sections) lies at the anterior symphysis of Meckel's cartilage. Despite obvious bleaching of the histological stain due to ageing (Figures 5a and 5b), grayscale VRs yield good results. In this developmental stage the embryonic dentary bone shows several trabeculae growing around the mandibular cartilage. A tooth develops distal to the dentary bone on the lateral side of the jaw (Figure 5c). Adjusting the transparency of the volume rendering allows the visualization of the different layers forming inside the tooth (Figure 5d). The traverse of a blood vessel as well as an underlying nerve cord is clearly visible along the whole rendered region.

## Discussion

### Benefits of volume rendering in visualizing light microscopical serial sections

To date, polygon-based surface rendering (SR) is still the only widely used method to present light microscopical serial section data three-dimensionally. In comparison volume rendering (VR) offers a number of crucial benefits for such data sets. Aligned image stacks based on serial sections can be readily visualized through VR for a quick overview of a whole specimen or an area of interest, whereas surface reconstructions always require either threshold-based or manual image segmentation prior to rendering. Manual image segmentation especially entails a considerable time effort, and the results are highly dependent on individual interpretation. In contrast, for VR the effort is significantly lower and results depend much less on interpretation. The effort of VR is especially negligible in image stacks already treated for SR. A combination of both rendering techniques could be even more valuable, and in fact this option is available in 3D software packages like Amira. In this context, it should be noted that a color inversion is not explicitly prerequisite to VR of grayscale image stacks, since it is possible to invert the artificial colormap (including the transparency function) during VR. Since the manual segmentation work for a surface reconstruction should always be done on the original non-inverted section images, the background of the aligned image stack should be white (avoiding black edge areas resulting from rotation and translation during image alignment in Amira can be easily achieved by changing the output value under the *AlignSlices *options menu to 255 (white, default value is 0: black)). In a combined use of VR and SR, strong visual emphasis can be given to selected structures like specific organ systems or tissues by manual segmentation and surface visualization, while the surrounding tissues and/or overall body shape is shown as a semi-transparent whole-view VR.

Probably the most significant benefit of VR as compared with SR lies in the complete preservation of three-dimensional grayscale or color image information during rendering. By image segmentation the image stack information is reduced to binary level (selected material vs. background or other material). Consequently, polygon-based surface rendering always entails a substantial loss of information (from at 8-bit/24-bit to 1-bit per segmented material). In contrast volume rendering potentially transfers the complete set of information contained in the section image stack into the 3D space, since every voxel is displayed according to its gray or color value just as in the original section image. Additionally, VRs allow visualizing structures that are too small or too thin for segmentation and SR, like thin muscle fibers, ciliated areas, and cell nuclei or nucleoli.

### General drawbacks and technical limitations in serial section VR

In visualizing serial physical sections via VR, time and labor demanded by the technical procedure of serial sectioning represent the main general drawback when compared with other 3D imaging methods [11]. Consequently we do not expect the presented method to be used for processing large numbers of samples (e.g. for mutant phenotyping, for which microCT, microMRI, and other tomographic techniques appear to be more suitable). It might rather be useful for the investigation and visualization of single specimens. This could be, for example, rare developmental stages or even type specimens (which are often kept in the form of serial sections).

The procedure of using serial sections to create 3D image stacks suitable for high-quality VR is not only time-consuming but also contains numerous potential error sources. While digital image acquisition and processing is done using permanent hardware setups and saved software settings, the protocols for producing histological sections can be sources of various artifacts. Streicher et al. [42] pointed out three main obstacles in the reconstruction of serial physical sections: misalignment, geometric distortion, and staining variation. They introduced the External Marker-based Automatic Congruencing concept (EMAC) to overcome these difficulties, a versatile approach that uses external markers for automatic image alignment and geometrical correction of distortion, and additionally balances staining variations. We want to explicitly stress that by applying EMAC or similar approaches, image stack quality could be even further optimized than in the protocols given in the present study. Nevertheless, our aim was to create easy and repeatable protocols that can be applied to different types of light microscopical sections, including historical and other already existing serial sections that obviously possess no external marker structures. In our approach, we use the *least-squares *algorithm of the Amira *AlignSlices *tool (for further details on image alignment see also [43]), which performs an automatic image registration by translation and rotation of adjacent slices. In our opinion, this algorithm yields excellent overall results, and in numerous cases, we were able to verify alignment results by a priori knowledge of the object's shape. However, problems in the alignment process could possibly arise if notable differences in stain bleaching occur at adjacent slides, since the *least-squares *algorithm seeks to minimize gray-value differences between corresponding points. Furthermore, different lighting conditions could cause similar problems.

Geometric inhomogeneities are largely negligible in resin sections, since they undergo only minimal distortions in terms of differences in stretching. Hence, high-quality VR can be performed right after image alignment without further image filtering or editing (as shown in Figure 1, black pathway). Although unfiltered image stacks based on resin sections usually yield very good results, in some cases a modest smoothing proved to be helpful for the final 3D image. Slight smoothing decreases the information of the original section images, but sometimes significantly eases perception of the final volume visualization while preserving almost all fine details. Distortions are much more of a problem with regard to paraffin wax sections, and significantly affect the quality of the 3D result. The presented 3D image filters are able to balance these distortions to a certain degree enabling high-quality VR also for paraffin wax sections (as already shown in Figures 3, 4c, d, and 5).

Staining variation across a series of sections is also a very important issue affecting the quality of the final 3D image. Dealing with historical material may be particularly difficult, because in old paraffin wax sections bleaching of histological stains represent a considerable problem. Thus original color VRs with adequate expression of the histological stain are sometimes unachievable (Figure 5). Nevertheless, VRs of grayscale section images will yield good results in the majority of cases. It seems necessary to mention that editing images of historical sections prior to the composition of the image stack will sometimes require more time and effort than will images of newly prepared section series. Standard image processing functions, such as auto-contrast and auto-level adjustments, yield good results in equalizing color or grayscale values, but would not work in every case.

Another resolution-limiting factor is section thickness. Three-dimensional image stacks constitute blocks (or 3D matrices) of volumetric pixels (voxels), and the edge-length of these voxels in the sectioning axis (z-axis) sets a limit on resolution in the 3D image. Hence, the accessible resolution of 3D image stacks made from semithin resin sections is comparatively high, since resins allow sectioning thickness to as low as 1 μm or even 0.5 μm. Although resolution along the sectioning axis is limited to approximately 0.5 μm, resolutions down to approximately 0.25 μm in the within-image axes (x- and y-axes) can allow even higher resolution in the final 3D image (voxel size = 0.25 μm × 0.25 μm × 0.5 μm). This enables visualizations on sub-cellular level (Figures 4a and 4b, note 1 μm sections) in an object size range between 100 μm and several millimeters. In image stacks based on paraffin wax sections the larger section thickness, typically 3 μm to 15 μm, yields a lower z-axial resolution. Consequently, we suggest that volume rendering of paraffin sections will offer the best results for objects from about 500 μm to several millimeters in size. Because of limits on section thickness and slight differences in staining, an image stack gained from light microscopical sections can never be completely isotropic. Consequently, every VR based on sections will contain some striated sectioning artifacts. These artifacts are especially visible when viewing the stack "edge-on," normal to the plane of sectioning (Additional file 1).

### Choosing suitable filter parameters

We want to point out that the filter parameters used here proved to be suitable for the presented examples and thus should be viewed as suggestions, not as prerequisites. Filter attributes such as kernel size, for example, must be chosen specifically for every stack, based on a trade-off between removing distortions and preserving detail. Consequently, the attributes given for described filters were found to be suited for the presented image stacks based on image stack resolution, magnitude of geometric distortions, and desired level of detail in the final rendering.

**Figure 6:**
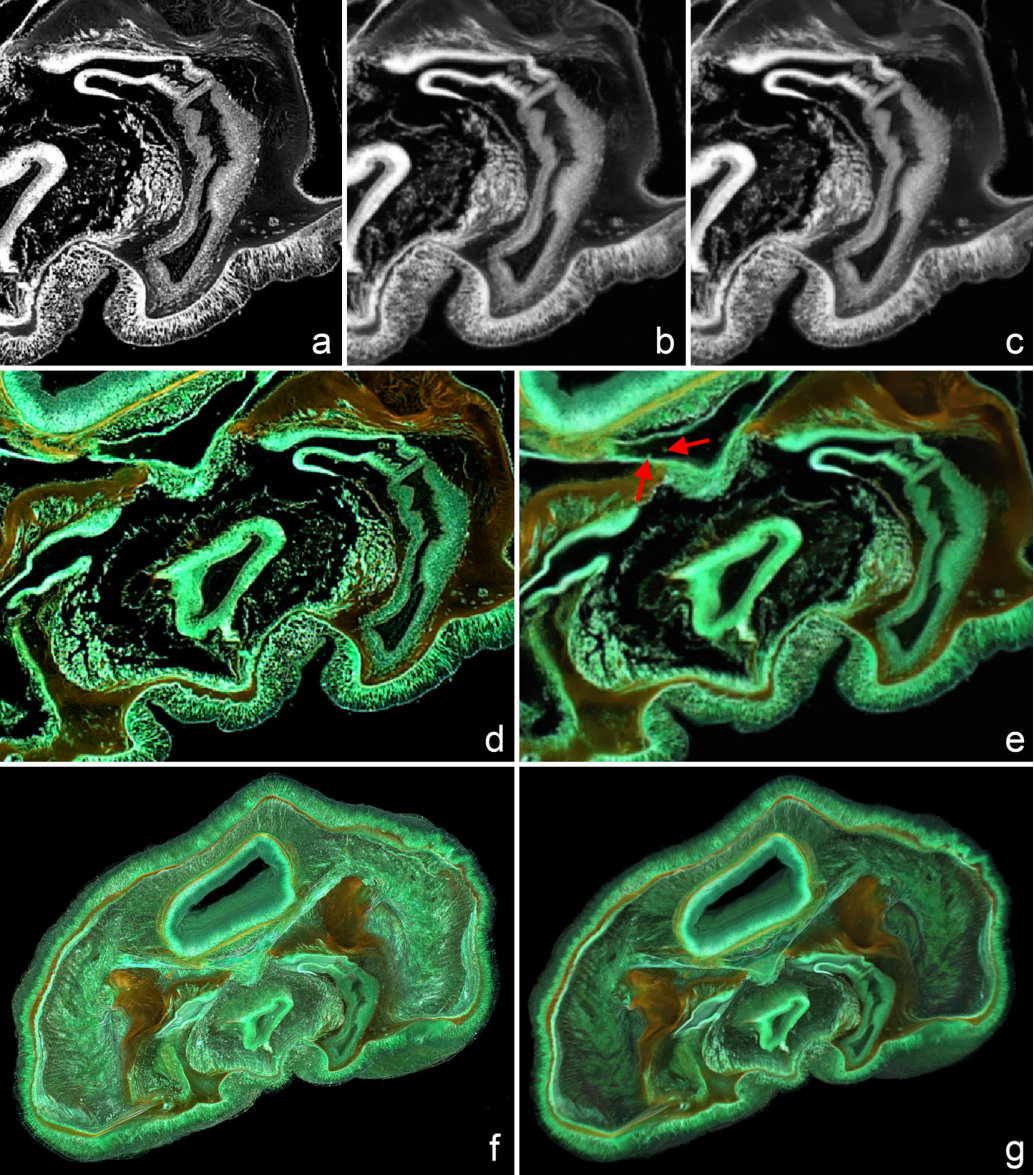
**Using digital image filters to increase the quality of volume rendering from paraffin wax sections**. (a) Unfiltered image stack of *Phoronis australis *sections: detail of a single slide from the unfiltered green channel. (b) First filtering step: detail of a single slide from the green channel after applying the Amira 3D Gauss filter (6 × 6 × 6 kernel, sigma = 1, 1, 1). (c) Second filtering step: detail of a single slide from the green channel after applying the Amira 3D edge-preserving-smoothing filter (contrast = 3.5, sigma = 3, step = 5, stop = 25) on the previously already Gauss-filtered stack. (d) Slide from the unfiltered RGBA stack. (e) Slide from the filtered RGBA stack after applying the two aforementioned filters (a-c). Note that structures as small as single blood cells remain to be distinguishable after both filtering steps (red arrows). (f) Volume rendering based on the unfiltered RGBA stack. Through the high level of very fine-scale detail, perception of the 3D structures is more difficult. (g) Volume rendering based on the filtered RGBA stack. Besides balancing geometric distortions, image filters decrease the amount of very fine-scale information and thus significantly ease perception of 3D structures.

We use the RGBA image stack of *Phoronis australis *(Figures 1, 4c, and 4d) to illustrate the effects of filters to the image stack in more detail. Since the stack is based on micrographs from paraffin wax sections in the first filtering step a rather big kernel (6 × 6 × 6) was chosen to smooth out geometric distortions, followed by an edge-preserving smoothing filter applied to each of the three color channels (Figures 1, 6a, b, and 6c). Alternatively, smaller kernels can be used if geometric distortions are less prominent. No exact minimum distance between structures can be given for these features to be still recognizable after smoothing, since this depends strongly on contrast between specific structures. However, in the *Phoronis australis *example structures as tiny as single blood cells (approximate size about 12 μm) can still be distinguished after smoothing (Figures 6d and 6e). Finally, one important function of the use of filters besides balancing geometric distortions is to ease perception of the rendering by removing very fine-scale details (Figures 6f and 6g).

### Choosing suitable colormaps and transparency functions

Choosing colormaps and transparency functions is crucial for what the final rendering should look like, especially for grayscale image stacks in which both color and transparency of voxels are assigned based on the same 8 bits of information. Before discussing the importance of colormaps and transparency functions in volume rendering it is again worth mentioning that color inversion of image stacks is not explicitly prerequisite for volume rendering in elaborate 3D software packages as Amira, since both colormaps and transparency functions can be adapted and inverted. Nevertheless, we generally recommend a color inversion since many volume viewers are designed for viewing tomographic data and thus require inverted data (object = bright, background = dark). In addition, default colormaps are commonly designed to view tomographic or confocal data and thus are inverted relative to light microscopical section images. Furthermore, we think that dark backgrounds and dark shades in rendering of colored image stacks strengthen the 3D impression of the object (for a comparison see also Figures 4c and 4d, as well as Additional files 2 and 3).

A number of standard default colormaps are included in rendering software packages (for Amira e.g. *glow*, *red*, *green*, *physics*), and the Amira *colormap *tool also allows creating specific colormaps value by value. The main aim in choosing a suitable colormap is to create a final rendering that (1) shows an overall well-contrasted image giving a strong 3D impression and (2) still allows one to distinguish details clearly (like e.g. cell nuclei). We think for rendering of biological tissue samples the *glow *colormap (which is also commonly used in visualizing confocal images) is highly suitable because it contains numerous colors (black, brownish, reddish, yellowish, white) in a wide contrast range from black to white. However, we modified this default map a little by cutting off the highest values to prevent the image from being too glary. Choosing a suitable transparency function is as important as choosing the right colormap. The Amira *Alpha curve *tool (embedded in the advanced colormap module) enables one to adjust the transparency function, starting from a linear function (gamma = 1) to softer (gamma > 1) or harder (gamma < 1) renderings.

**Figure 7:**
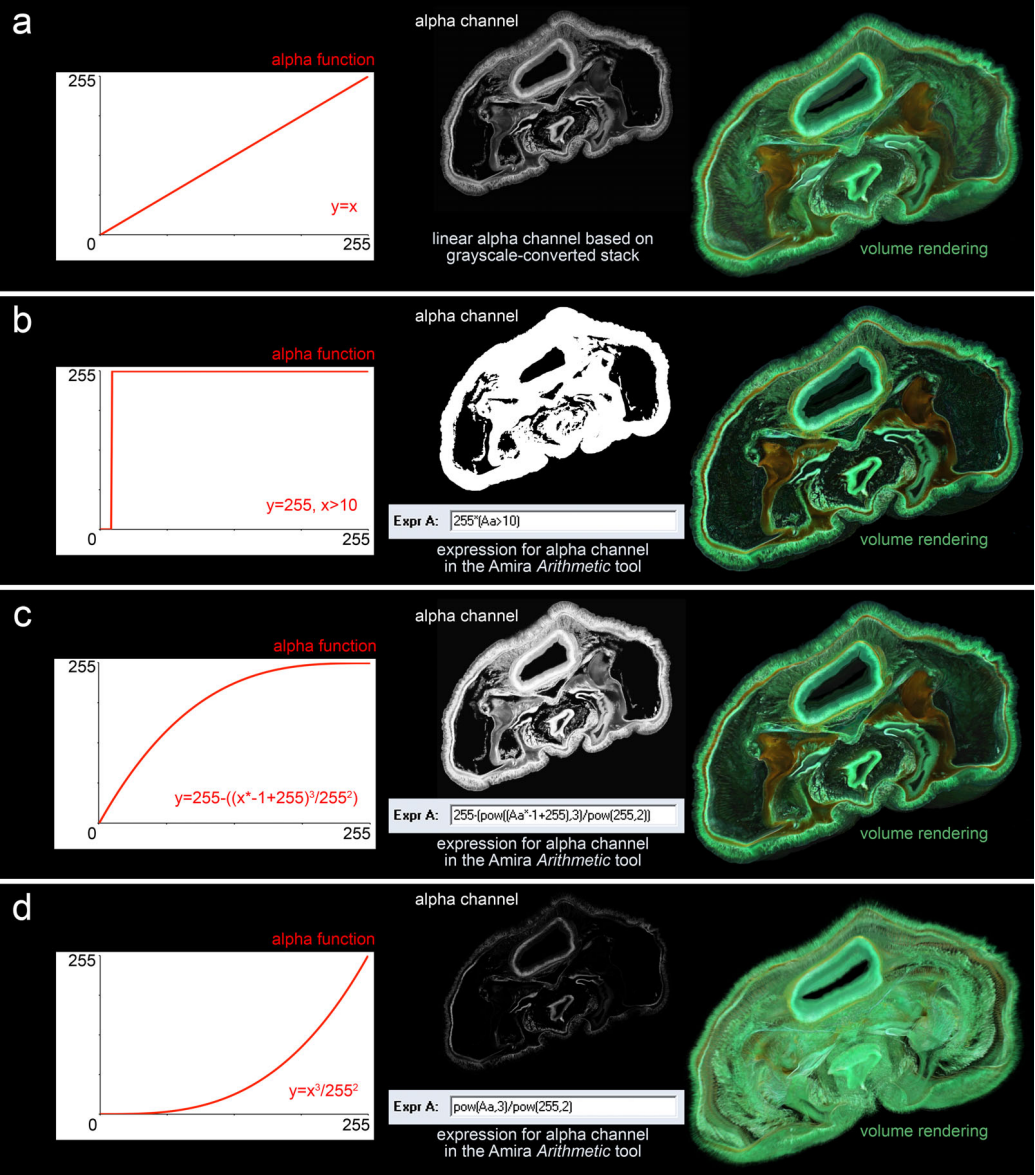
**Various alternative transparency functions for volume rendering based on colored image stacks**. Different transparency functions offer various options in volume rendering of colored image stacks. (a) A linear transparency function based on grayscale-conversion of the image stack. (b) A threshold-based (threshold = 10) transparency function yielding a very hard volume rendering with completely opaque object voxels and completely transparent background voxels. The Amira *Arithmetic *module is used to transform the alpha channel of the RGBA stack using the threshold expression "Expr A: 255*(Aa > 10)", while the three color channels remain unchanged ("Expr R: Ar", "Expr G: Ag", "Expr B: Ab"). (c) A quadratic transparency function with decreasing slope of the curve yielding a rendering that is harder than using a linear function, but obviously softer as the threshold-based rendering. In the demonstrated example we again used the Amira *Arithmetic *module with the alpha channel expression "Expr A: 255-(pow((Aa*-1+255),3)/pow(255,2))". Alternatively one could also use a logarithmic function to obtain similar results. (d) A quadratic transparency function with increasing slope of the curve yielding a rendering that is much softer than using a linear function. In the demonstrated example we used the expression "Expr A: pow(Aa,3)/pow(255,2)". Alternatively one could also use an exponential function to obtain similar results.

Rendering of color image stacks is different because color information for rendering is stored for each voxel and consequently no colormap is required. The appearance of the volume rendering, however, can be adjusted and optimized by using various kinds of transparency functions. Based on the linear transparency function (Figure 7a, also already seen in Figure 4) calculated from color information following the standard weighting grayscale conversion formula "gray value = 0.3 R + 0.59 G + 0.11 B", the alpha channel can be adjusted using the Amira *Arithmetic *tool. With this tool the linear alpha channel can be transformed by a user-specified mathematical expression, while the three color channels remain unchanged ("Expr R: Ar", "Expr G: Ag", "Expr B: Ab"). Using a threshold-based transparency function ("Expr A: 255*(Aa > x)") yields a very hard volume rendering with completely opaque object voxels (Figure 7b). Quadratic transparency functions with decreasing slope of the curve using any equation of the form "Expr A: 255-(pow((Aa*-1+255), x)/pow(255, x-1))" yield renderings that are harder than those using a linear function, but obviously softer than the threshold-based rendering (Figure 7c). With increasing powers the curve gets steeper; one could also use logarithmic functions to obtain similar results. Quadratic transparency functions with increasing slope of the curve yield renderings that are much softer than with a linear function (Figure 7d). Similar to the last example, one can use any equation of the form "Expr A: pow(Aa, x)/pow(255, x-1)". Again, with increasing powers the curve gets steeper; one could also use an exponential function to achieve a similar effect.

### Range of application

We have introduced VR protocols for the visualization software Amira for different embedding media and histological stains. Various other 3D software packages also include VR and image filter tools that are suitable for VR of image stacks based on serial section images, such as Imaris (Bitplane Scientific Solutions, Zurich, Switzerland), OsiriX (The OsiriX Foundation, Geneva, Switzerland), VG Studio MAX (Volume Graphics GmbH, Heidelberg, Germany), ImageJ (developed at the National Institutes of Health), Voxx (Indiana Center for Biological Medicine, University of Indiana), and various others (for a review on microscopy-orientated software packages see also [44]). These image stacks contain three-dimensional raw data acquired by histological preparation, image capturing, and alignment. Although VR algorithms only visualize information already stored in serial section images and do not generate novel data, 3D visualizations offer clear advantages in presenting and communicating morphological data. Thus, we see a special value and benefit of the presented protocols in creating 3D images that can be used for morphological databases focusing on development (such as the Edinburgh Mouse Atlas [45,46] or FishNet [47]), as well as for teaching purposes, and for textbooks. Small and complex objects like invertebrate larvae, such as echinoderm plutei, phoronid actinotrochae, and numerous others represent some of the most fascinating objects for visualization of complex morphological features. In our opinion full-color true-volume data resulting from section series would substantially improve the presentation of respective specimens in diverse oral and printed presentations.

As given above, VR can be utilized in visualizing not only new but also historical serial sections. Much of our present knowledge of microanatomy and development was gained during the golden age of histology in the 19th and early 20th centuries. Today many scientific institutions still house a substantial number of such section series. Often these are of superb quality containing material that is rare today or completely unavailable due to extinction or habitat destruction.

Molecular patterns from sections could also be visualized in 3D with volumetric reconstructions from light microscopical sections. The method of GeneEMAC [48,49] represents a standard protocol for SR of 3D molecular patterns from histological sections, based on whole mount in-situ hybridized embryos and using automatic image capturing methods along with automatic threshold segmentation for visualization of gene expression signals. These fine-scale threshold-segmented renderings of molecular patterns are actually very similar to true VR in showing the spatial distribution of a signal, but thresholding still yields a loss of original gray- or color-information in segmented voxels. VR visualizations of gene expression patterns could add a new level of information in understanding three-dimensional molecular patterns, since biomolecules generally occur in gradients rather than discrete areas, and true volume renderings would even allow a 3D visualization of continuous gradients.

### Related 3D imaging methods

Volume rendering based on serial sections fills an important gap in the field of three-dimensional micro-anatomical visualization methods. A major strength of the method is the two-sided approach comprising high quality 3D imaging on the one hand and original histological sections for reliable ground-truth in tissue identification on the other hand. We emphasize that by exploiting the tissue specificities of different histological stains, VR from light microscopic serial sections differs fundamentally from any other 3D imaging method.

In general, the resolution of a single image decreases with increasing specimen (and field of view) size, and thus the physical size of the specimen directly affects image stack resolution in VR. This is true for serial section reconstructions just as for all other 3D imaging methods. However, this can be overcome in the case of light microscopical section series. A single series can be utilized to create several image stacks from different object areas (i.e. different regions of view) and at different resolutions. This could be done by acquiring section images with different optical magnifications, for example, with 40× magnification for a general 3D specimen overview, and 400× magnification for 3D visualization of selected organs or tissues. From light microscopical sections, useful 3D visualizations can be obtained covering a huge object size range between approximately 100 μm to at least several centimeters, yielding resolutions higher than in most currently used tomographic 3D imaging methods (microCT, microMRI, or OPT). Confocal microscopy methods (like CLSM) produce 3D images of extremely high resolution, but are limited to very small specimens [13].

Another important benefit of the presented protocols lies in the broad color range of standard and highly specific histological stains. By using the whole spectrum of visible light for information transfer, these stains offer rich contrast and give various options for discrimination of many different tissues at the same time (e.g. bone, hyaline cartilage, teeth, nerves, and blood vessels as shown in Figure 5). Using VR this color information can be directly transferred into 3D space (see Figures 1 and 4). For comparison, microCT produces only grayscale images of tissue densities. Although a number of x-ray contrasting agents for soft tissues were recently reported [2], no highly tissue-selective stains are known for this technique to date. Imaging systems based on fluorescent probes (OPT, CLSM) require samples that are mostly transparent for visible light and therefore cannot take advantage of the whole range of histological tissue stains. The relatively recent projection-tomographic method of OPT produces volumetric images showing protein and nucleic acid localization in whole tissues. Thus OPT has occupied its own important niche, molecular probes imaged at the scale of whole embryos and tissues [11]. The section-tomographic method of CLSM also takes special advantage of highly specific fluorescent dyes. Its range of application includes selective staining of tissues like muscle fibers or nerves [13], as well as detection of local gene expression. Using conventional non-fluorescent histological stains, three-dimensional tissue discrimination of fine structures cannot be readily achieved as in fluorescence-based imaging methods. This is because specific histological stains (as well as fluorescent dyes) are mostly restricted to use on paraffin wax sections, which are limited with respect to low z-axial resolution. We see a special value in a combined use of specific visualization using fluorescence-imaging methods on the one hand and VRs based on serial sections on the other hand, since histological stains allow contrasting of various different tissues and consequently to evaluate and visualize the spatial relationships between tissues.

## Conclusions

The presented set of protocols combines classical histological techniques with modern 3D image filtering and visualization methods. As such, VR of serial sections enables high-resolution 3D visualizations of small and complex biological specimens using standard equipment and software available to most labs. At present, mainly snapshots and video clips are used for publishing VR data. Recently, Ruthensteiner and Heß [50] reported a method for embedding 3D models based on SR in digital publications like PDFs. This kind of published 3D data is extremely valuable and those files including embedded polygon-surfaces do not exceed a file size of a few megabytes. Because of the notably larger file sizes of volume files, it remains to be seen whether embedding of VR data as described by Barnes and Fluke [51] will become accessible and suitable for VRs of biological specimens during the next few years.

## Competing interests

The authors declare that they have no competing interests.

## Authors' contributions

SH developed protocols and drafted the manuscript. TS carried out most parts of practical laboratory work. BDM contributed to data handling as well as optimization of 3D rendering and presentation. All authors contributed to the content and writing of this paper and approved the final version of the manuscript.

## Supplementary Material

Additional file 1 **Volume rendering using artificial colors**. This video shows an artificial-color volume rendering based on resin sections of the *Cristatella mucedo *specimen shown in Figure 2. Click here for file

Additional file 2 **Volume rendering using the inverted histological colors**. This video shows an inverted full-color volume rendering based on paraffin sections of the *Phoronis australis *specimen shown in Figures 3, 4, 6, and 7. Inversion of colors prior to rendering is necessary to achieve total transparency in the background. Click here for file

Additional file 3 **Full-color micro-morphology**. Re-inversion of colors (as shown in Figure 1) in a snapshot or video (in this case Additional file 2) yields a 3D visualization in the real colors of the histological stain. Click here for file
